# How to approach patients and families at the end of life

**DOI:** 10.4102/safp.v66i1.5916

**Published:** 2024-04-24

**Authors:** Maggie De Swardt, Rene Krause, Louis S. Jenkins

**Affiliations:** 1 Department of Family, Community and Emergency Care, Faculty of Health Sciences, University of Cape Town, Cape Town, South Africa; 2 Department of Family and Emergency Medicine, Faculty of Medicine and Health Sciences, Stellenbosch University, Cape Town, South Africa; 3 Primary Health Care Directorate, Department of Family, Community and Emergency Care, Faculty of Health Sciences, University of Cape Town, Cape Town, South Africa; 4 Department of Family and Emergency Medicine, Western Cape Department of Health, George Hospital, George, South Africa

**Keywords:** palliative care, end-of-life, multi-professional teams, symptoms, difficult conversation

## Abstract

Healthcare practitioners are regularly faced with treating patients at the end of their life, and this can be very daunting. This article hopes to help the practitioner have an approach to managing end-of-life care that makes it less distressing. The symptoms at the end-of-life include delirium and/or agitation, breathing changes, skin changes, sleeping more, decrease in need for food and drink, incontinence, and increased secretions. These symptoms are discussed and practical ways of management are given. The article further discusses how to approach the difficult conversation with the family and gives guidance as to what needs to be discussed. A number of tips are discussed on how to prepare the family to handle a death at home. It is essential to look at coping mechanisms and selfcare for practitioners dealing with end-of-life care as the death of a patient not only affects the family but also the practitioner.

## Introduction

Healthcare practitioners are faced with situations where a patient in their care is experiencing the last few days of their life. This time during the end of life may be experienced with apprehension and anxiety by everyone involved in the dying person’s life if one is not prepared. Various palliative care resources and hospice support structures are available to provide support, help and understanding during this time.^1,2^ While palliative medicine is now a well-established discipline, many healthcare practitioners remain uncertain or untrained on how to approach patients and their families at the end-of-life. Healthcare practitioners need skills in communication, especially how to break bad news and contain a patient and family, to manage common symptoms, offer psychosocial and spiritual support, incorporate community resources, and how to be aware of their own responses in dealing with suffering and death.^3^ The Health Professions Council of South Africa (HPCSA) published a useful booklet called ‘Ethical Guidelines on Palliative Care’, which helps healthcare practitioners understand how personal and professional values and ethical principles influence end-of-life care.^4^

Context is very important and highly variable in South Africa. Increasingly, people with advanced diseases are preferring to die at home.^5,6^ Home visits are not typically conducted by most healthcare practitioners, which is a skill in itself. Some patients end up in hospices, hospital wards, community health centres or tuberculosis (TB) hospitals at the end of their lives. Young people with diseases such as TB or acquired immunodeficiency syndrome (AIDS), and sometimes children with advanced terminal diseases, are a reality in South Africa. Understanding the roles of every member of the multi-professional palliative care team, community health workers, and family members is paramount. Listening with empathy and shared decision-making with the family and carers are crucial. Taking time, being comfortable with uncertainty and allowing periods of silence, are all part of the process.^3^

## Symptoms at the end-of-life

Various symptoms appear during this time. Not all symptoms mentioned here appear at the same time and some may never appear at all. All the symptoms described indicate that the body is preparing itself for the final stage of life (see Table 1).^7,8,9^ In general, unnecessary investigations and invasive interventions should be avoided, medications should be rationalised, and changing baseline organ functions should be considered.

**TABLE 1:** Symptoms and responses at the end of a person’s life.

No	Symptom	Non-pharmacological management	Pharmacological or medical management
1	The person may gradually become more sleepy during the day and at times will be difficult to arouse.	The best time to communicate with the person is when they seem most alert, but communicate at all times. It is important to prevent bedsores: turn the patient regularly and do pressure care. The use of a sheepskin, convoluted foam, or a ripple, bubble or eggshell mattress can help, if available.	Consider review of all medications that may cause sedation. Reduce the dose of medications as organ functions are reduced.
2	Delirium or agitation The person may become increasingly confused about time, place, and identity of family and friends who are normally familiar to them. It is important to note that pain and anxiety in children in the terminal phase may present as stoic behaviour and often missed as they do not express it the same way adults do.	Talk calmly and confidently with the confused person to reassure them and to prevent startling or frightening them. Using orientation tools and identifying yourself by name, may lessen the confusion. Remember: anxiety, pain, full bladder and constipation can contribute to restlessness and agitation. Treat these first.	Only consider adding medication if patient is distressed, hallucinating or a danger to self or others. The aim is not to sedate the patient, but to make the patient more comfortable. Low dose benzodiazepine, e.g. Lorazepam 0.5 mg – 1 mg prn or an antipsychotic (depending on availability) e.g. Haloperidol 0.5 mg – 1 mg up to 2.5 mg – 10 mg /24 h po or sc (or imi as last resort) OR Risperidone 0.5 mg – 1 mg nocte po prn OR Olanzapine 2.5 mg nocte prn po
3	There may be a decreased need for food and drink.	Reassure the family that loss of appetite is normal at the end of life. Attempting to feed a patient who is unable to swallow causes stress. Try moist swabs, fine mist spray or tiny amounts of crushed ice if the patient experiences thirst. Applying lip balm to dry lips is an option. Remember the patient is not dying because he or she is not eating. The patient is dying; that is why he or she is not eating.	They may appear dehydrated, because of loss of skin elasticity, but do not need a ‘drip’ at this stage. Do not give nasogastric tube feeding. It does not prevent aspiration and may cause aspiration and is uncomfortable. It also increases secretions, oedema and can cause nausea and vomiting. Hypodermoclysis is a possibility where a dying process is extremely prolonged or thirst is a problem.
4	As blood circulation slows down, the arms and legs may become cool to the touch and the underside of the body may become darker in colour.	If the person reports feeling cold, use one or two blankets to keep them comfortable. Avoid too many bedclothes or an electric blanket as this may lead to overheating and increased restlessness. Autonomic dysfunction causes an unstable temperature and patients are usually most comfortable with a simple sheet.	-
5	Loss of bladder control may occur.	Use incontinence pads, nappies or linen savers to protect the patient and bedding. Change these regularly to prevent excoriation and other skin issues.	Anticholinergics do not help.
6	Urine output may decrease as death becomes closer.	Reassure the family. Accept as part of normal end-of-life journey. Explain that urine may be dark or light in colour.	No need for blood investigations or intravenous (IV) fluids.
7	Involuntary movements may occur when the person is very close to death.	Reassure the family. Accept as part of normal end-of-life journey. Myoclonic movements could be an adverse effect from accumulation of opioid metabolites and toxicity because of changing renal function.	Consider adding low dose benzodiazepine, e.g. Lorazepam or Clonazepam 0.5 mg – 2 mg 6–8 hourly sublingually, orally, or subcutaneously.
8	Saliva and mucus may collect in the throat as the person’s cough or swallowing reflex diminishes. This sometimes causes a noise, which can sound concerning.	This is usually not distressing to the patient. Do not suction the patient’s mouth or airways and rather reposition the patient. Turn them on their sides, have a cloth available and clean frequently. Physiotherapist can also help positioning and clearing of secretions.	Medications such as anticholinergics can help to dry up noisy secretions. For example, hyoscine butylbromide 20 mg 6–hourly, orally or subcutaneously. OR Glycopyrrolate 0.2 mg–0.4 mg q 4 hourly sc OR Atropine drops in the mouth prn
9	Dyspnoea or irregular breathing patterns may occur, with seconds to minutes between breaths.	Reassure the family. Accept as part of normal end-of-life journey. If the person appears to be in respiratory distress, have an open window, or a fan gently ventilating the person.	Consider low dose Mist. Morphine 0.5 ml–1 ml prn to 4 hourly sublingual OR Fentanyl patch 12.5 ug – 25 ug if no other interventions are possible. Consider Lorazepam 1 mg sublingual prn for anxiety. The subcutaneous or rectal route is best at this stage.
10	Vision may decrease slightly.	When vision decreases, be guided by the patient, and maybe provide light in the room, particularly at night. The use of a night light may also help.	-
11	Hearing is thought to be one of the last senses to be lost.	Do not assume the person cannot hear. Let them know that you are there – this will give them support and comfort. There are six important things to say to someone at this time, which can make it easier for them to gently ‘let go’^7^ I’m sorry I forgive you Thank you I love you It’s ok to die, we will be ok Goodbye	-

*Source*: Hospice Palliative Care Association of South Africa. Clinical Guidelines 2012 [homepage on the Internet]. [cited 2024 Jan 24]. Available from: https://apcc.org.za/wp-content/uploads/2020/04/HPCA_Clinical_guidelines_2012-1.pdfAPCC; Hospice United Kingdom. What to expect when someone is in the last few days of life [homepage on the Internet]. [cited 2024 Jan 24]. Available from: https://www.hospiceuk.org/information-and-support/death-and-dying-what-expect/last-few-days; Wilcock A, Howard P, Charlesworth S, editors. Palliative Care Formulary. London: Pharmaceutical Press; 2020

## Having the difficult conversation with the family

The discussion with the family starts early in the palliative phase and does not happen just before dying. The sooner the patient gets opportunity to express his or her wishes to the family, the better and easier the practitioner can navigate the last part of the journey of life with the family. Before speaking to the family, ensure you are speaking to the correct family members. The patient is the best person to identify who should be spoken to. If the patient is unable to communicate, ensure you are speaking to the next of kin as indicated in the records. Prepare for the meeting by ensuring you and the whole treating team are informed and agree that the patient is nearing the end of life and are in agreement about the future care. Ensure all conversations are held in a quiet private space. Decisions about whether the patient should be included in the conversation should be determined by the patient’s wishes and physical and emotional state.^2^ As far as possible, maintain patient autonomy, also depending on how they engaged with their health and the health system in the past.

Start the conversation with formal introductions and affirming confidentiality. Open the conversation by asking the family what they understand about what is happening to the person. Their understanding of the person’s condition and their emotional response will determine what must be said, the extent and, the level of emotional support that is required. If they are not aware of the seriousness of the patient’s condition a breaking bad news conversation should be followed.^10^ Many times, families are aware of the seriousness of the patient’s condition and your response to their narrative should be to affirm the emotional response to their understanding of the patient’s condition. If the family are aware that the patient is very sick but has not realised that the patient is dying, the doctor must provide a warning shot and inform the family that the patient is dying (unfortunately, sadly I have to inform you that I am worried that Mr X is at the end of his life or dying). Using the words ‘I am worried’ softens the conversation. Then ensure you follow this with silence and enough time to contain the family’s emotions. Most families will enquire ‘how long’ and at this point you can inform them that no doctor knows the exact time for certain but from what is currently happening you will estimate short days, weeks, among others. Do not give a number and again inform the family that you may be wrong. However, if the family have certain tasks to be completed, for example, another family who wants to visit, or cultural and religious rituals that can be done before life ends, encourage them to rather do this earlier than later. Be sensitive to cultural practices like fetching the soul if the person does not pass away in his or her family home. Ask the family if they are ready to have a further discussion or whether they want to first have time to be alone.^10^

The next part of the conversation is planning the end-of-life care. This conversation should follow an ask, listen, tell and respond to the emotions format. Ask about what is important to the patient and family. Ask about worries and concerns. Inform them about what care is currently in place, for example, pain relief and comfort care measures. Enquire about the place of care and whether they want to take the patient home. If the family wishes to take the patient home, do an appropriate community referral, education about what to expect and what to do when the patient passes away. Ensure any vulnerable family members have been identified and adequate psycho-social care and bereavement care is in place, for example, elderly spouses or young children. Enquire about spiritual care and link to the family’s own or hospital spiritual resources. Offer to link the family to bereavement support. Ensure the family have easy access to the patient and that all relevant family members have been informed.^11^

After the conversation, have enough time for questions and share with them how to get in contact with your team for follow-up conversations. Document the conversation.^10^

## Tips on how to prepare the family for a death at home

- The family member(s) may need someone to be with them. Think about who that would be and keep their telephone number on hand.
- Explain to the family the signs of death: no breathing, no pulse and/or heartbeat; loss of control of bladder and bowel; no response; eyes become dull and glazed, might be half open and dries out; jaw relaxes and mouth slightly open; body becomes cold, skin colour changes and body will eventually become stiff.
- After the passing of the patient the family can phone the ambulance, who will do the death declaration and then they can contact the undertaker.
- The family need to have the patient’s ID document or passport available for the ambulance and undertaker.

There is no need to call the ambulance or undertaker immediately. It is often helpful for the family to have time with the person who has died before the undertaker arrives.

Patients who choose to die in hospital, need a slightly different approach (not covered in this article).

## Be aware of your own feelings and coping mechanisms

Rituals like a minute of silence after the death of a patient to ground yourself and staff involved, lighting a candle, saying a prayer or having a memory wall can be very helpful, as it is normal to be affected by a patient’s death. In difficult cases, a debrief session with the family and also all staff involved afterwards and even counselling or just talking about it, can also make a big difference. Take care of your own mental health and seek advice or help if not coping. There may be a sense of relief with the family or practitioner that the suffering of the patient is over, which in turn may invoke a sense of guilt. The death of a patient often affects the family and even the physician many months later. Be aware of this.

## Conclusion

Palliative care at the end-of-life needs specific skills to address the various patient symptoms and relational family and personal dynamics. Hopefully, this brief article will contribute to the growing body of excellent resources available.
